# A two-column process for bispecific antibody purification based on MabSelect VL resin’s strong byproduct removal capability

**DOI:** 10.14440/jbm.2025.0106

**Published:** 2024-12-12

**Authors:** Wanyuan Dong, Penglong Zhang, Di Wu, Yan Wan, Yifeng Li

**Affiliations:** Downstream Process Development (DSPD), WuXi Biologics, Shanghai, 200131, China

**Keywords:** Aggregate, Bispecific antibody, MabSelect VL, Protein A, Protein L, Three-column process, Two-column process

## Abstract

**Background:**

Protein L-conjugated resins are affinity media that bind to the variable region of the kappa light chain (LC) and have been used for initial product capture in the downstream processing of full-length antibodies and antibody fragments. Previous studies, including ours, have demonstrated that Protein L chromatography effectively separated various byproducts generated during the production of bispecific antibodies (bsAbs), including half-antibody, homodimer, LC-missing species, and aggregates. Cytiva recently launched its second-generation Protein L resin, MabSelect VL, which offers significantly improved binding capacity compared to its predecessor, Capto L.

**Objective:**

This study aimed to explore the feasibility of developing a two-column process, which includes MabSelect VL capture step and a polishing step, for purification of complex antibody molecules.

**Methods:**

We employed two bsAb cases to demonstrate that MabSelect VL’s enhanced byproduct removal capability allows for a potential two-column purification process.

**Results:**

For both bsAbs, the developed two-column process yielded a product with quality attributes comparable to those obtained using the traditional three-column process.

**Conclusion:**

The MabSelect VL-based two-column process can be successfully applied to bsAb purification. In addition, it should also be feasible with regular monoclonal antibodies, whose purification is generally less challenging than that of bsAbs. By reducing the downstream process from three columns to two columns, significant savings in terms of time, labor, and materials can be achieved.

## 1. Introduction

Protein L is a bacterial surface protein that interacts with the variable region of the antibody kappa light chain (LC).^1^ It consists of four or five highly homologous, consecutive antibody-binding domains.^2^,^3^ Each Protein L domain has two distinct LC binding sites and can bind two fragment antigen-binding regions simultaneously.^4^,^5^ Commercially available Protein L-conjugated resins, including Toyopearl AF-rProtein L-650F from Tosoh, Capto L/MabSelect VL from Cytiva, and KanCap L from Kaneka, are commonly used for initial product capture in the downstream processing of full-length antibodies and antibody fragments. During the production of asymmetric bispecific antibodies (bsAbs), certain product-related byproducts contain fewer Protein L binding sites than the target product. For example, half-antibody and LC-missing species bind Protein L monovalently, while the target bsAb binds to it bivalently. The difference in binding valency between the product and byproducts forms the basis for their separation through Protein L chromatography.^6^-^8^ In addition to separating species with different binding valencies, Protein L chromatography can even distinguish between antibody species that possess the same number of binding sites.^9^ We have previously shown that different antibodies bind to Protein L columns with varying strengths, even when all contain two Protein L binding sites. As the two half-antibodies of asymmetric bsAbs are derived from two different parental monoclonal antibodies (mAbs), this unique property of Protein L (i.e., its ability to exhibit variable binding strengths toward different antibodies) renders it a promising tool for separating the target heterodimer from homodimer byproducts.^9^ Furthermore, Protein L chromatography has been shown to effectively separate monomers from aggregates,^6^,^10^ a process that can be further enhanced by adding salt to the elution buffer.^10^

Despite its strong capability for removing byproducts and aggregates, Protein L affinity chromatography is used less frequently than Protein A chromatography for product capture in downstream processing. A major factor that prevents the broader application of Protein L-conjugated resins is that they generally have a binding capacity lower than that of Protein A affinity resins. Recently, Cytiva introduced a second-generation Protein L resin, MabSelect VL, which shows a significantly improved binding capacity.^11^ This development opens up new possibilities for the more widespread application of Protein L chromatography. In this study, we explored the possibility of developing a two-column process using MabSelect VL’s strong byproduct/aggregate removal capability, in the context of two bsAb cases. The two bsAbs (A and B) adopted in symmetric and asymmetric formats, respectively. For bsAb A, the culture harvest contained a high percentage (23.3%) of aggregates. With bsAb B, the culture harvest had half-antibody, homodimers, LC-missing species (¾ antibody), and aggregates. For both bsAbs, a three-column process, including Protein A capture followed by two polishing steps, had previously been developed to reduce both process- and product-related impurities to acceptable levels. In both cases, when MabSelect VL was utilized for capture instead of Protein A, it effectively removed significant amounts of product-related impurities. As a result, post-MabSelect VL, only a single polishing step was required to achieve the same impurity reduction as the original three-column process. The successful application of the MabSelect VL-based two-column process for bsAbs implies that this approach is also feasible with regular mAbs, whose purification is typically less challenging. By leveraging the strong byproduct removal capability of MabSelect VL, the traditional three-column process in downstream purification could potentially be simplified to a two-column process, leading to significant time and cost savings.

## 2. Materials and methods

### 2.1. Materials

Ammonium sulfate, ethanol, sodium caprylate, sodium acetate trihydrate, sodium chloride, sodium hydroxide, and tris(hydroxymethyl)aminomethane were purchased from Merck (Germany). Acetic acid was procured from J.T. Baker (USA). MabSelect VL (200 mL, product code: 17542002), Capto adhere ImpRes (25 mL, product code: 17371501), Tricorn 5/150 column (0.5 cm I.D.), and XK 26 column (2.6 cm I.D.) were obtained from Cytiva (Sweden). EZ 6.6/300 columns (6.6 mm I.D.) were from Omnifit (UK). The TSK G3000SW_XL_ stainless steel column (7.8 × 300 mm) was bought from TOSOH (Japan). POROS XS (25 mL, catalog number: 4404339), N-Ethylmaleimide, 20× 2-(N-morpholino)ethanesulfonic acid (MES) running buffer, and 4× lithium dodecyl sulfate (LDS) sample buffer came from Thermo Fisher Scientific (USA). Precast SurePAGE 4–12% gradient Bis-Tris gels were from GenScript (China). Precision Plus Protein Unstained Standards (1 mL, catalog number: 1610363) were purchased from Bio-Rad Laboratories (USA). The WXB CHO-K1 host cell proteins (HCP) enzyme-linked immunosorbent assay (ELISA) Kit was obtained from WuXi Biologics (China). X0HC and A1HC depth filters were bought from Millipore (Germany). The two bsAbs used in the current study were expressed in stably transfected CHO-K1 cells cultured in HyClone ActiPro medium, supplemented with Cell Boost 7a and 7b (feeding supplements from Cytiva, Sweden). The cell culture was maintained for 14 days before harvest.

### 2.2. Equipment

An ÄKTA pure 150 system equipped with Unicorn software version 7.8 (Cytiva, Sweden) was used for column chromatography. A Tecan Freedom EVO 200 (Switzerland) was employed in the load condition screening studies. pH and conductivity were measured using SevenExcellence S470 pH/Conductivity Meter (Mettler-Toledo, USA). Protein concentration was determined using NanoDrop 2000 spectrophotometer (Thermo Fisher Scientific, USA). An Agilent 1260 liquid chromatography system (Agilent Technologies, USA) was used for size-exclusion chromatography-high performance liquid chromatographic (SEC-HPLC) analysis. A LabChip GXII Touch HT instrument (PerkinElmer, USA) was applied for Caliper analysis. The plate for HCP quantitation was read on an Infinite 200 PRO plate reader (Tecan, Switzerland).

### 2.3. MabSelect VL chromatography

A 0.5 cm diameter column with a 15.0 cm bed height and a 2.6 cm diameter column with a 20.0 cm bed height were individually packed with MabSelect VL resin. The column volumes (CVs) were approximately 2.9 and 106.2 mL, respectively. The columns were loaded with clarified culture harvest at 30 mg and 40 mg of protein per mL of resin for bsAb A and bsAb B, respectively. The runs were conducted under linear or stepwise pH gradient elution (see Table 1 for detailed protocols). For the small and large columns, the system was operated at flow rates of 180 and 240 cm/h, respectively, with a residence time of 5 min.

**Table 1:** Key parameters and buffer conditions for MabSelect VL chromatography

Molecule	Step	CV	Composition
BsAb A	Equilibration	5/3^a^	50 mM Tris, HOAc, 150 mM NaCl, pH 7.4
	Load	NA^b^	Clarified harvest
	Wash 1	5/3^a^	50 mM Tris, HOAc, 150 mM NaCl, pH 7.4
	Wash 2	5/3^a^	20 mM NaOAc-HOAc, 4/5^c^/6 mM (NH_4_)_2_SO_4_, pH 5.5
	Elution	As needed	20 mM NaOAc-HOAc, 4/5^c^/6 mM (NH_4_)_2_SO_4_, pH 3.4
	Strip	3	120 mM HOAc
	Sanitization	3	100 mM NaOH
BsAb B	Equilibration	5/3^a^	50 mM Tris, HOAc, 150 mM NaCl, pH 7.4
	Load	NA^b^	Clarified harvest
	Wash 1	5/3^a^	50 mM Tris, HOAc, 150 mM NaCl, pH 7.4
	Wash 2	5/3^a^	50 mM NaOAc-HOAc, 5 mM NaCl, pH 5.0^d^
			30 mM NaOAc-HOAc, pH 4.8^e^
			50 mM Tris, HOAc, 0.2 M sodium caprylate, pH 7.4^f^
	Wash 3	5/3^a^	50 mM Tris, HOAc, 25 mM NaCl, pH 7.4^e,f^
	Elution	As needed	A: 50 mM NaOAc-HOAc, 5 mM NaCl, pH 5.0^d^ B: 50 mM NaOAc-HOAc, 25 mM NaCl, pH 3.0^d^
			30 mM NaOAc-HOAc, 25 mM NaCl, pH 3.8^e^/3.9^e,f^/4.0^e^
	Strip	3	120 mM HOAc
	Sanitization	3	100 mM NaOH

Abbreviations: bsAb: Bispecific antibody; CV: Column volume; HOAc: Acetic acid; NaCl: Sodium chloride; NaOAc: Sodium acetate; NaOH: Sodium hydroxide; (NH_4_)_2_SO_4_: Ammonium sulfate. Notes:

a For small and large columns, respectively.

b Not applicable.

c For the finalized protocol.

d For linear pH gradient elution (100% B was reached over 20 column volume).

e For stepwise elution.

f For the finalized stepwise elution protocol.

### 2.4. Capto adhere ImpRes loading condition screening for bispecific antibody A

For bsAb A, Capto adhere ImpRes mixed-mode resin was used as the polishing step post capture, and its loading conditions were screened using robotic liquid handling unit (Freedom EVO 200, Tecan, Switzerland) with a 96-well filter plate. A total of 20 μL of Capto adhere ImpRes resin was added to individual wells of the plate, which were then equilibrated to the selected sodium chloride (NaCl) concentrations (0, 125, 250, or 500 mM) and pH values (5.0, 6.0, 7.0, or 8.0). Subsequently, 123 μL of MabSelect VL eluate (with conductivity and pH adjusted to the corresponding values) was added to each well (loading density: 50 mg/mL). The plate was shaken at 1,100 revolutions per minute (rpm) for 60 min. After shaking, the supernatant from each well was collected, and the concentration of unbound protein was determined by measuring UV absorbance at 280 nm. Flow-through yield was calculated by determining the concentration ratio of flow-through to the load.

### 2.5. Capto adhere ImpRes chromatography for bispecific antibody A

A 0.66 cm diameter column was packed with Capto adhere ImpRes resin to a bed height of 15.8 cm, resulting in a CV of approximately 5.4 mL. The column was loaded with MabSelect VL eluate following intermediate depth filtration (int. DF), with pH and NaCl concentrations adjusted to 6.0 and 250 mM, respectively, at a loading density of 50 or 100 mg/mL. The column was then washed with 50 mM sodium acetate (NaOAc)-acetic acid (HOAc), 250 mM NaCl, pH 6.0. For additional information, please refer to the protocol in Table 2. The system was operated at a flow rate of 190 cm/h (residence time: 5 min).

**Table 2:** Key parameters for Capto adhere ImpRes and POROS XS chromatography, used as post-MabSelect VL polishing steps for bispecific antibodies A and B, respectively

Molecule	Step	CV	Composition
BsAb A	Equilibration	5	50 mM NaOAc-HOAc, 250 mM NaCl, pH 6.0
	Load	NA^a^	MabSelect VL eluate post-int. DF
	Wash	5	50 mM NaOAc-HOAc, 250 mM NaCl, pH 6.0
	Strip	3	500 mM Arg-HCl
	Sanitization	3	1 M NaOH
BsAb B	Equilibration	5	50 mM NaOAc-HOAc, pH 5.5
	Load	NA^a^	MabSelect VL eluate post-int. DF
	Wash	5	50 mM NaOAc-HOAc, pH 5.5
	Elution	20+5^b^	A: 50 mM NaOAc-HOAc, pH 5.5 B: 50 mM NaOAc-HOAc, 1 M NaCl, pH 5.5
	Strip	3	50 mM NaOAc-HOAc, 1 M NaCl, pH 5.5
	Sanitization	3	1 M NaOH

Abbreviations: Arg-HCl: Arginine hydrochloride; bsAb: Bispecific antibody; CV: Column volume; HOAc: Acetic acid; int. DF: Intermediate depth filtration; NaCl: Sodium chloride; NaOAc: Sodium acetate; NaOH: Sodium hydroxide. Notes:

a Not applicable.

b 0–25% B over 20 CV, followed by an additional 5 CV of 25% B.

### 2.6. POROS XS cation exchange (CEX) chromatography for bispecific antibody B

POROS XS resin was packed into a 0.5 cm diameter column with a 15.0 cm bed height (CV: ~2.9 mL). The column was loaded with Protein L eluate following intermediate DF, with pH adjusted to 5.5, at a loading density of 30 mg/mL. For wash and elution details, please refer to the protocol in Table 2. The system was operated at a flow rate of 180 cm/h (residence time: 5 min).

### 2.7. Non-reducing sodium dodecyl sulfate-polyacrylamide gel electrophoresis (SDS-PAGE)

Non-reducing SDS-PAGE was performed using precast SurePAGE 4–12% gradient Bis-Tris gels. Sample loading buffer (4× LDS) and gel running buffer (20× MES) were obtained from Thermo Fisher Scientific. Samples were heated at 75°C for 5 min. Equal protein amounts (~0.5 μg/well) were loaded onto the gels. Electrophoresis was carried out at 120 V for 120 min. Gels were stained and destained using eStain LG protein staining system from GenScript (China).

### 2.8. Size-exclusion chromatography-high performance liquid chromatography

Size-exclusion chromatography-high performance liquid chromatography analysis was performed using an Agilent 1260 liquid chromatography instrument with a TSK G3000SW_XL_ stainless steel column (7.8 × 300 mm). A total of 100 μg of sample was injected per run. The mobile phase consisted of 50 mM sodium phosphate and 300 mM NaCl at pH 6.8. Each sample was eluted isocratically for 20 min at a flow rate of 1.0 mL/min. Protein elution was monitored by UV absorbance at 280 nm.

### 2.9. Caliper analysis under non-reducing conditions

Samples for analysis were prepared by mixing with LDS sample buffer, N-Ethylmaleimide, and pure water. The samples were incubated at 70°C for 10 min, then analyzed on a LabChip GXII Touch HT instrument (2 μg of sample injected per run).

### 2.10. Host cell protein measurement

Host cell protein levels were measured by using the WXB CHO-K1 HCP ELISA Kit, as per the manufacturer’s instructions. The detection range was 3–100 ng/mL. The samples were serially diluted to ensure the measurements remained within the calibration range. Absorbance was measured at 450 nm (test) and 650 nm (reference) on the Infinite 200 PRO plate reader.

## 3. Results and discussion

### 3.1. MabSelect VL chromatography for capture

**Figure 1:**
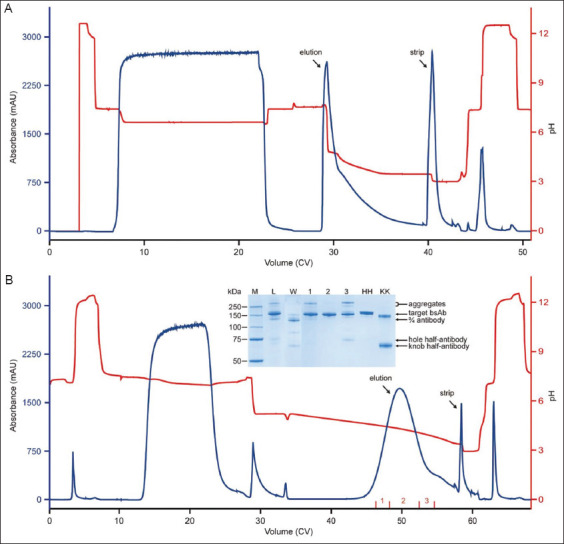
MabSelect VL chromatograms of runs conducted to capture (A) bispecific antibody (bsAb) A and (B) bsAb B. For (A) and (B), the runs were conducted using stepwise and linear pH gradient elution, respectively. Inset: Sodium dodecyl sulfate-polyacrylamide gel electrophoresis (SDS-PAGE) analysis of relevant fractions. Notes: M: Protein markers; L: Load; W: Wash; Lanes 1–3: Elution fractions 1–3; HH: Pure hole-hole homodimer used as a reference; KK: Pure knob-knob homodimer used as a reference. A portion of the KK homodimer was formed through non-covalent interactions and disassociated into half-antibodies under the denaturing conditions used in the SDS-PAGE. On the gel, the HH and KK homodimers migrated marginally more slowly and faster than the target bsAb, respectively. Bands corresponding to the various byproducts are labeled.

For bsAb A, the primary challenge was the removal of aggregates. According to SEC-HPLC data, the culture harvest contained 23.3% aggregates. Protein L chromatography has been shown to be effective in removing aggregates.^6^,^10^ We previously found that, under pH stepwise elution conditions, adding a small amount of ammonium sulfate to the elution buffer further improved monomer-aggregate separation (authors’ unpublished observation). In the current study, using a 3 mL column, we first screened three different ammonium sulfate concentrations (4, 5, and 6 mM), by following the protocol outlined in Table 1. The results are summarized in Table 3. The SEC-HPLC purity of the eluate (89.8–91.9%) was significantly improved compared to that previously achieved by Protein A chromatography (79.8%). Based on the data, higher ammonium sulfate concentration slightly improved purity but compromised yield. Therefore, the intermediate concentration of 5 mM was selected to balance quality and yield. A run was conducted using a 106 mL column according to the finalized protocol, and the chromatogram is shown in Figure 1A. For the eluate from this run, the monomer content was 92.0% (as determined by SEC-HPLC).

**Table 3:** Results of ammonium sulfate concentration screening for aggregate removal

Run	(NH_4_)_2_SO_4_ concentration (mM)	Fraction	SEC-HPLC (%) HMW/Monomer/LMW^a^	Mass (%)	Monomer yield (%)^b^
1	4	Eluate	9.5/89.8/0.7	73.5	91.2
		Strip	NA^c^	14.9	NA^c^
2	5	Eluate	8.0/91.5/0.5	69.0	87.2
		Strip	NA^c^	19.4	NA^c^
3	6	Eluate	7.4/91.9/0.7	64.0	81.2
		Strip	NA^c^	26.7	NA^c^

Abbreviations: (NH_4_)_2_SO_4_: Ammonium sulfate; SEC-HPLC: Size-exclusion chromatography-high performance liquid chromatography. Notes:

a High-molecular-weight species, monomer, and low-molecular-weight species.

b Monomer yield=(monomer purity in eluate×mass)/monomer purity in load.

c Not applicable.

For bsAb B, its byproduct profile was more complex than that of bsAb A. In addition to aggregates, the culture harvest contained half-antibody, homodimers, and LC-missing species, which are common for bsAbs adopting asymmetric format. When the culture harvest was processed by Protein A chromatography, SEC-HPLC and non-reduced Caliper analysis of the eluate indicated that the main peak contents accounted for 88.2% and 85.0%, respectively, suggesting that most of the product-related impurities remained. We previously reported that half-antibody and LC-missing species bound more weakly than the product to a Protein L-conjugated resin, as they contain fewer binding sites and can be removed by an appropriate washing step.^7^,^8^ The two types of homodimers can bind more feebly or strongly than the target bsAb, depending on the properties of the corresponding parental mAb.^9^ Aggregates consistently bind more tightly than the monomer.^6^,^10^ In this study, when a run was conducted under linear pH gradient elution (Figure 1B for the chromatogram), half-antibody, knob-knob homodimer, and LC-missing species were eluted during the intermediate pH wash, as indicated by SDS-PAGE analysis of the corresponding fraction (Figure 1B, inset). The SDS-PAGE also showed that hole-hole homodimer and aggregates were enriched in the late-eluting fractions (Figure 1B, inset). Based on the linear pH gradient results, three pH values (3.8, 3.9, and 4.0) were screened while developing the stepwise gradient elution. Eventually, pH 3.9 was selected to balance product quality and yield. In addition, pH 4.8 was chosen for the intermediate wash. The washing step was further optimized by adding 0.2 M sodium caprylate to the wash buffer, a known enhancer of HCP clearance.^12^-^14^ After optimization, the HCP level in the eluate was reduced from 2,428 ng/mg (without optimization) to 938 ng/mg, while effectively removing weakly bound byproducts. Finally, a run was performed following the finalized protocol using a larger column (CV: 106 mL). The main species content in its eluate, as determined by SEC-HPLC and non-reduced Caliper analysis, was 95.2% and 98.1%, respectively, which represented a significant improvement compared to the corresponding values obtained from Protein A eluate.

### 3.2. Mixed-mode or CEX chromatography for polishing

**Figure 2:**
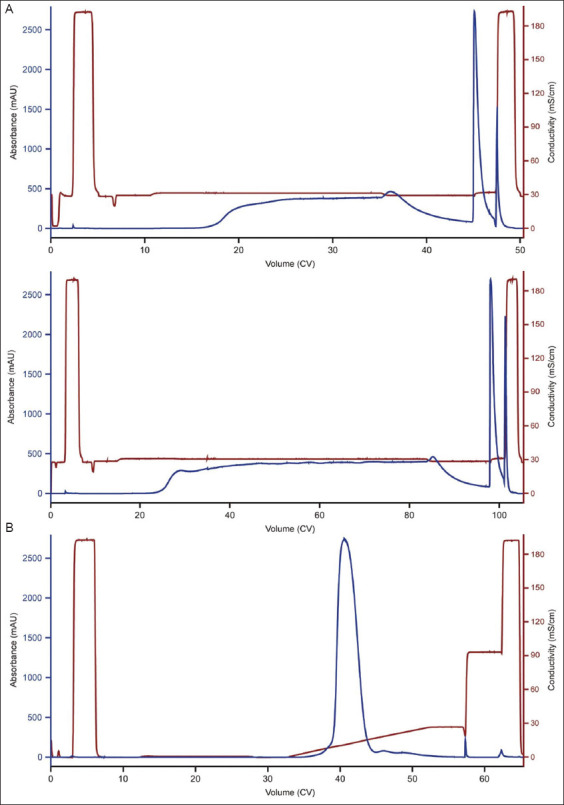
Mixed-mode and cation exchange (CEX) chromatography for the polishing step of bispecific antibodies (bsAbs) A and B. (A) Capto adhere ImpRes chromatogram of runs conducted for bsAb A under flow-through mode. The top and bottom panels show runs conducted at loading densities of 50 and 100 mg/mL, respectively. (B) CEX chromatogram of a run conducted for bsAb B under linear conductivity gradient elution. Abbreviation: CV: Column volume.

For bsAb A, following MabSelect VL affinity chromatography, the sample still contained approximately 8% aggregates. When anion exchange (AEX) chromatography was conducted under flow-through mode (chromatogram not shown) for polishing, it provided limited aggregate clearance (data not shown). Capto adhere ImpRes is a mixed-mode resin from Cytiva that combines AEX and hydrophobic interactions, and it is known to provide effective aggregate clearance under flow-through mode.^15^-^17^ Therefore, this resin was selected for the polishing step following MabSelect VL capture. The high-throughput screening was used to evaluate different loading pH and conductivity conditions that favor flow-through mode. Results for several selected loading conditions are shown in Table 4. While higher pH and conductivity improved aggregate removal, yield was compromised under these conditions. Ultimately, a loading condition of 50 mM NaOAc-HOAc, 250 mM NaCl, pH 6.0 was selected and verified using a 5.4 mL Capto adhere ImpRes column under two loading densities (i.e., 50 and 100 mg/mL; Figure 2A for the chromatograms). SEC-HPLC analysis of the flow-through indicated that monomer content was improved to 99.1% and 98.6% under low and high loading densities, respectively. The corresponding yield was 73.8% and 82.7%, respectively.

**Table 4:** Results for selected loading conditions in Capto adhere ImpRes chromatography

Condition	Yield (%)	SEC-HPLC (%) HMW/Monomer/LMW^a^
pH 5.0, 125 mM NaCl	91.2	8.9/90.7/0.3
pH 5.0, 250 mM NaCl	70.5	5.2/94.3/0.5
pH 6.0, 0 mM NaCl	73.0	7.8/91.8/0.4
pH 6.0, 125 mM NaCl	61.2	4.1/95.4/0.5
pH 6.0, 250 mM NaCl	42.9	2.0/97.1/0.9

Abbreviations: NaCl: Sodium chloride; SEC-HPLC: Size-exclusion chromatography-high performance liquid chromatography. Note:

a High-molecular-weight species, monomer, and low-molecular-weight species.

For bsAb B, CEX chromatography was used as the polishing step following MabSelect VL capture. CEX was preferred over AEX because it provided better HCP clearance in this case. Under linear conductivity gradient elution (Figure 2B for the chromatogram), SEC-HPLC and Caliper analyses of the eluate showed further improvement in purity, reaching 99.6% and 99.0%, respectively. In downstream processing, AEX chromatography is typically a critical step for viral clearance, as it generally provides more effective virus removal than CEX chromatography.^18^-^21^ In this case, while CEX provided adequate HCP and byproduct clearance as the sole polishing step, it may need further optimization to improve viral clearance.

### 3.3. Comparison between two-column and three-column processes

For both bsAbs, a three-column process was previously developed, consisting of Protein A capture and ensuing two polishing steps. Given that Protein A chromatography typically lacks the capability to separate aggregates and other product-related impurities, the removal of these byproducts primarily relies on the two polishing steps. When MabSelect VL was used to replace Protein A for capture, its strong byproduct removal capability significantly reduced the purification burden on the polishing steps, making it possible to achieve the same level of impurity reduction with only one polishing step. In addition, as the two-column process spreads the purification burden across multiple steps rather than relying on a single step, it is more robust. A comparison between the two-column and three-column processes is detailed in Table 5. While both processes delivered the final product with comparable quality attributes, the two-column process resulted in a slightly higher overall yield.

**Table 5:** Comparison between two-column and three-column processes

BsAb	Process	Step	Resin/depth filter	Loading density (g/L)	SEC-HPLC (%) HMW/Monomer/LMW^a^	Non-reduced Caliper (%)	HCP (ng/mg)	Step yield (%)	Total yield (%)
A	Two-column	AC	MabSelect VL	30	7.6/92.0/0.4	NA^b^	4628	67.1	50.5
		Int. DF	X0HC	827 (g/m^2^)	8.9/90.6/0.5	NA^b^	<94	96.2	
		MMC	Capto adhere ImpRes	50	0.5/99.1/0.4	NA^b^	47	73.8	
				100	1.0/98.6/0.4	NA^b^	50	82.7	
	Three-column	AC	MabSelect SuRe LX	30	20.0/79.8/0.1	NA^b^	5375	86.7	49.6
		Int. DF	X0HC	825 (g/m^2^)	19.0/80.9/0.2	NA^b^	<132	95.5	
		AEX	POROS 50HQ	77	18.9/80.9/0.1	NA^b^	<82	99.1	
		MMC	Diamond MMC Mustang	30	0.6/99.3/0.1	NA^b^	<47	60.4	
B	Two-column	AC	MabSelect VL	40	4.7/95.2/0.1	98.1	938	60.1	49.2
		Int. DF	A1HC	877 (g/m^2^)	3.8/96.1/0.1	98.5	565	95.9	
		CEX	POROS XS	30	0.4/99.6/0.0	99.0	64	85.4	
	Three-column	AC	MabSelect SuRe LX	40	9.2/88.2/2.5	85.0	4157	89.1	48.3
		Int. DF	A1HC	1131 (g/m^2^)	8.1/89.6/2.3	85.8	417	94.5	
		MMC	Capto MMC ImpRes	80	5.9/92.2/1.8	95.5	310	77.5	
		AEX	POROS 50HQ	40	0.7/99.3/ND^c^	99.2	59	74.0	

Abbreviations: AC: Affinity chromatography; AEX: Anion exchange; BsAb: Bispecific antibody; CEX: Cation exchange; HCP: Host cell protein; Int. DF: Intermediate depth filtration; MMC: Mixed-mode chromatography; SEC-HPLC: Size-exclusion chromatography-high performance liquid chromatography.

Notes:

a High-molecular-weight species, monomer, and low-molecular-weight species,

b Not applicable,

c Not detected.

## 4. Conclusion

Protein L-conjugated resins have shown strong capabilities for eliminating byproducts and aggregates; however, previous commercial products have suffered from low binding capacities. Recently, Cytiva launched MabSelect VL, a new Protein L resin that exhibits significantly improved binding capacity compared to its predecessor, Capto L. The introduction of this new resin paves the way for a more extensive application of Protein L chromatography in the downstream processing of antibodies. In the current study, we demonstrated that, with the help of MabSelect VL’s strong impurity-removing capability, a two-column process is possible even for bsAbs with complex byproduct profiles. Given that the downstream processing of mAbs is generally less challenging than that of bsAbs; our results also suggest that the MabSelect VL-based two-column process is viable for most mAbs. While additional case studies are warranted to further explore the general applicability of the two-column process in downstream processing, the current study clearly showed that MabSelect VL, with its strong byproduct removal capability, makes the development of a two-column purification process both easier and more feasible than before.

## Data Availability

The data and supporting information are available within this article.

## References

[1] Björck L. Protein L. A novel bacterial cell wall protein with affinity for Ig L chains. J Immunol. 1988;140(4):1194-1197. 3125250

[2] Kastern W Sjöbring U Björck L. Structure of peptostreptococcal protein L and identification of a repeated immunoglobulin light chain-binding domain. J Biol Chem. 1992;267(18):12820-12825. 1618782

[3] Murphy JP Duggleby CJ Atkinson MA Trowern AR Atkinson T Goward CR. The functional units of a peptostreptococcal protein L. Mol Microbiol. 1994;12(6):911-920. 10.1111/j.1365-2958.1994.tb01079.x. 7934898

[4] Graille M Stura EA Housden NG. Complex between *Peptostreptococcus magnus* protein L and a human antibody reveals structural convergence in the interaction modes of Fab binding proteins. Structure. 2001;9(8):679-687. 10.1016/s0969-2126(01)00630-x. 11587642

[5] Paloni M Cavallotti C. Molecular modeling of the interaction of protein L with antibodies. ACS Omega. 2017;2(10):6464-6472. 10.1021/acsomega.7b01123. 31457247 PMC6645367

[6] Chen C Wakabayashi T Muraoka M. Controlled conductivity at low pH in Protein L chromatography enables separation of bispecific and other antibody formats by their binding valency. MAbs. 2019;11(4):632-638. 10.1080/19420862.2019.1583996. 30898021 PMC6601544

[7] Wang Y Chen X Wang Y Li Y. Removing a difficult-to-separate byproduct by Capto L affinity chromatography during the purification of a WuXiBody-based bispecific antibody. Protein Expr Purif. 2020;175:105713. 10.1016/j.pep.2020.105713. 32738439

[8] Chen W Zhang T Wan Y Li Y. Assessing four subdomain-specific affinity resins'capability to separate half-antibody from intact bispecific antibody. Protein Expr Purif. 2022;198:106124. 10.1016/j.pep.2022.106124. 35661701

[9] Chen X Wang Y Wang Y Li Y. Protein L chromatography:A useful tool for monitoring/separating homodimers during the purification of IgG-like asymmetric bispecific antibodies. Protein Expr Purif. 2020;175:105711. 10.1016/j.pep.2020.105711. 32738435

[10] Chen SW Tan D Yang YS Zhang W. Investigation of the effect of salt additives in Protein L affinity chromatography for the purification of tandem single-chain variable fragment bispecific antibodies. MAbs. 2020;12(1):1718440. 10.1080/19420862.2020.1718440. 31983280 PMC6999846

[11] . Cytiva. MabSelect VL Affinity Chromatography Resin, Data File, CY26149. Marlborough, MA:Cytiva. 2022.

[12] Aboulaich N Chung WK Thompson JH Larkin C Robbins D Zhu M. A novel approach to monitor clearance of host cell proteins associated with monoclonal antibodies. Biotechnol Prog. 2014;30(5):1114-1124. 10.1002/btpr.1948. 25044920 PMC4415537

[13] Chollangi S Parker R Singh N Li Y Borys M Li Z. Development of robust antibody purification by optimizing protein-A chromatography in combination with precipitation methodologies. Biotechnol Bioeng. 2015;112(11):2292-2304. 10.1002/bit.25639. 25950654

[14] Cui T Chi B Thompson JH Kasali T Sellick C Turner R. Cathepsin D:Removal strategy on protein A chromatography, near real time monitoring and characterisation during monoclonal antibody production. J Biotechnol. 2019;305:51-60. 10.1016/j.jbiotec.2019.08.013. 31442501

[15] . Capto adhere ImpRes Available from: https://cdn.cytivalifesciences.com/api/public/content/digi-16466-pdf.

[16] Gao D Wanga LL Lin DQ Yao SJ. Evaluating antibody monomer separation from associated aggregates using mixed-mode chromatography. J Chromatogr A. 2013;1294:70-75. 10.1016/j.chroma.2013.04.018. 23639130

[17] Chen SW Hoi KM Mahfut FB Yang Y Zhang W. Effective flow-through polishing strategies for knob-into-hole bispecific antibodies. Bioresour Bioprocess. 2022;9(1):98. 10.1186/s40643-022-00590-8. 38647877 PMC10992779

[18] Cai K Anderson J Orchard JD Afdahl CD Dickson M Li Y. Virus removal robustness of ion exchange chromatography. Biologicals. 2019;58:28-34. 10.1016/j.biologicals.2019.01.004. 30661901

[19] Strauss DM Gorrell J Plancarte M Blank GS Chen Q Yang B. Anion exchange chromatography provides a robust, predictable process to ensure viral safety of biotechnology products. Biotechnol Bioeng. 2009;102(1):168-175. 10.1002/bit.22051. 18683259

[20] Connell-Crowley L Nguyen T Bach J. Cation exchange chromatography provides effective retrovirus clearance for antibody purification processes. Biotechnol Bioeng. 2012;109(1):157-165. 10.1002/bit.23300. 21837666

[21] Miesegaes GR Lute S Strauss DM. Monoclonal antibody capture and viral clearance by cation exchange chromatography. Biotechnol Bioeng. 2012;109(8):2048-2058. 10.1002/bit.24480. 22488719

